# Unsatisfactory gene transfer into bone-resorbing osteoclasts with liposomal transfection systems

**DOI:** 10.1186/1477-5751-4-5

**Published:** 2005-08-29

**Authors:** Tiina Laitala-Leinonen

**Affiliations:** 1 Institute of Biomedicine, Department of Anatomy, University of Turku, Turku, Finland

## Abstract

**Background:**

Bone-resorbing osteoclasts are multinucleated cells that are formed via fusion of their hematopoietic stem cells. Many of the details of osteoclast formation, activation and motility remain unsolved. Therefore, there is an interest among bone biologists to transfect the terminally differentiated osteoclasts and follow their responses to the transgenes *in vitro*. Severe difficulties in transfecting the large, adherent osteoclasts have been encountered, however, making the use of modern cell biology tools in osteoclast research challenging. Transfection of mature osteoclasts by non-viral gene transfer systems has not been reported.

**Results:**

We have systematically screened the usefulness of several commercial DNA transfection systems in human osteoclasts and their mononuclear precursor cell cultures, and compared transfection efficacy to adenoviral DNA transfection. None of the liposome-based or endosome disruption-inducing systems could induce EGFP-actin expression in terminally differentiated osteoclasts. Instead, a massive cell death by apoptosis was found with all concentrations and liposome/DNA-ratios tested. Best transfection efficiencies were obtained by adenoviral gene delivery. Marginal DNA transfection was obtained by just adding the DNA to the cell culture medium. When bone marrow-derived CD34-positive precursor cells were transfected, some GFP-expression was found at the latest 24 h after transfection. Large numbers of apoptotic cells were found and those cells that remained alive, failed to form osteoclasts when cultured in the presence of RANKL and M-CSF, key regulators of osteoclast formation. In comparison, adenoviral gene delivery resulted in the transfection of CD34-positive cells that remained GFP-positive for up to 5 days and allowed osteoclast formation.

**Conclusion:**

Osteoclasts and their precursors are sensitive to liposomal transfection systems, which induce osteoclast apoptosis. Gene transfer to mononuclear osteoclast precursors or differentiated osteoclasts was not possible with any of the commercial transfection systems tested. Osteoclasts are non-dividing, adherent cells that are difficult to grow as confluent cultures, which may explain problems with transfection reagents. Large numbers of α_v_β_3 _integrin on the osteoclast surface allows adenovirus endocytosis and infection proceeds in dividing and non-dividing cells efficiently. Viral gene delivery is therefore currently the method of choice for osteoclast transfection.

## Background

Osteoclasts are bone-resorbing cells that are highly polarized when physiologically active [1]. Their mononuclear precursors are hematopoietic in origin, and remain non-adherent in culture until they differentiate further from the multipotent cell lineage [2,3]. Monocytes, macrophages and osteoclasts derive from the same precursor cells [4]. Multinuclear osteoclasts are formed by fusion of their committed mononuclear precursor cells and RANKL is the major growth factor inducing osteoclast formation [5]. Osteoclast morphology and activity is highly dependent on the matrix that they are cultured on, bone being their natural substrate. Mature osteoclasts undergo several cycles of activation and inactivation, where bone is resorbed in the active state and cells migrate in the resting state. Eventually, the cells die apoptotically and, *in vivo*, new bone formation by osteoblastic cells takes place to fill the resorption lacuna.

Cell transfection is used in biomedical research to study the role of individual gene products *in vitro *or *in vivo*. Viral and non-viral gene transfer systems are available from several suppliers, and several cell lines and primary cells can efficiently be transfected [6,7]. Physiological barriers, including the plasma membrane, still cause transfection difficulties with distinct cell types. Cell-surface glycosaminoglycans inhibit transfection *in vitro *[8], suggesting that efficient gene transfer is as a sum of many positively affecting parameters. Inside cells, DNA needs to escape from the endosomes before their maturation into lysosomes [9]. Cell-specific targeting of gene transfer particles would also be beneficial, and manipulating the gene transfer complexes by adding targeting proteins or peptides is currently under research [10].

When plasmid DNA is transfected to cells, it needs to be transported to the nucleus to reach the transcription machinery [11,12]. Nuclear transport may be achieved either during mitosis when the nuclear membrane becomes disrupted or by transport through the nuclear pores. Transfection of non-dividing cells may be obtained by activating nuclear uptake by inserting nuclear localization signals into the transgene [13,14].

Adenoviral gene transfer into osteoclasts has been shown to work well [15]. This is probably due to the numerous α_v_β_3 _integrin receptors that are located on the osteoclast plasma membrane [16]. Reports describing non-viral transfection on mature, adherent osteoclasts have not been found. There are also reports describing transfection of macrophages, like RAW264.7, that have after non-viral gene transfer been induced to form multinuclear giant cells [17]. It still remains controversial, however, whether these cells are polykaryons or truly osteoclasts capable of bone resorption. Due to a wish to study osteoclast migration and bone matrix removal in a more physiological context, we cultured osteoclasts and their early mononuclear precursors on bone and used these cultures for transfection. Earlier work in our laboratory suggested that other conventional transfection methods like calcium phosphate, DEAE-Dextran, electroporation, scrape-loading and hypotonic shock cannot be used. In the current paper we present data on the unsuccessful use of liposomal systems in the transfection of mature human osteoclasts and their mononuclear precursors *in vitro*.

## Results

### Transfection reagent-DNA ratio

Transfection reagents have specific reagent-to-DNA ratios that affect transfection efficiency and toxicity. In order to determine which ratios to use in the following experiments, we decided to test three ratios. On the basis of the morphological analysis of the cells, one test ratio was chosen for further analysis. Although disappointing at this stage, a more detailed study was continued to determine whether decreasing incubation time after transfection would allow transgene expression.

### Apoptosis index

**Figure 1:**
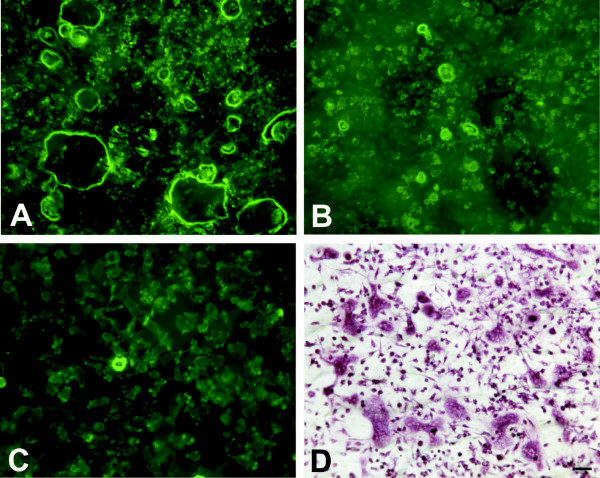
**Visualization of actin rings and TRACP-positive cells in osteoclast cultures. **Osteoclasts were differentiated in the presence of RANKL, M-CSF and TGF-β1 for 7 days, followed by fixation and staining of actin rings (a-c) and TRACP (d). Baseline control is shown in a and d, and adenovirus-infected cells 4 h post infection are shown in b. A typical view of the cells incubated 2 h with transfection reagents and 4 h in fresh medium is shown in c.

**Figure 2:**
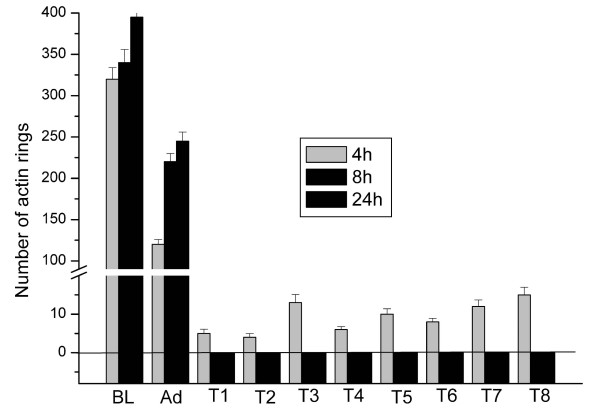
**Number of actin rings in osteoclast cultures. **Cells were treated with transfection reagents for 2 h, followed by culture for 4 h, 8, or 24 h. Cells were stained with phalloidin and number of actin rings was counted to quantitate actively resorbing osteoclasts. BL, baseline with no additions; Ad, adenoviral infection of GFP; T1-T8, transfection reagents as shown in Tables 1 and 2. ANOVA: p < 0,001

**Figure 3:**
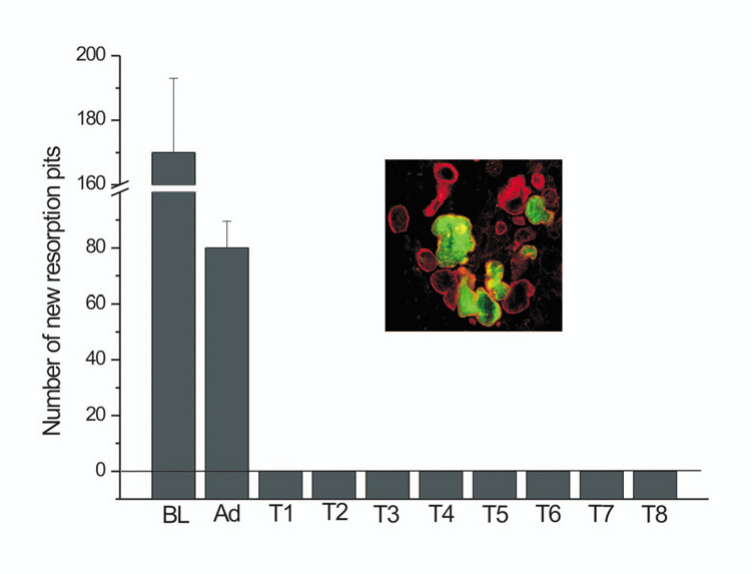
**Number of new resorption pits in osteoclast cultures. **Bone slices were biotinylated before cells were treated with transfection reagents for 2 h, followed by culture for 4 h, 8, or 24 h. Biotinylated resorption pits were visualized with FITC-labelled streptavidin and all resorption pits were stained with TRITC-WGA lectin. Resorption occurring after transfection was determined as pits emitting only red fluorescence. BL, baseline with no additions; Ad, adenoviral infection of GFP; T1-T8, transfection reagents as shown in Tables 1 and 2. The baseline control shown in the insert shows the staining pattern of the resorption pits before transfection (green) and overall resorption activity during the whole culture period (red). Yellow colour determines areas where both fluorochromes overlap. ANOVA: p < 0,001

**Figure 4:**
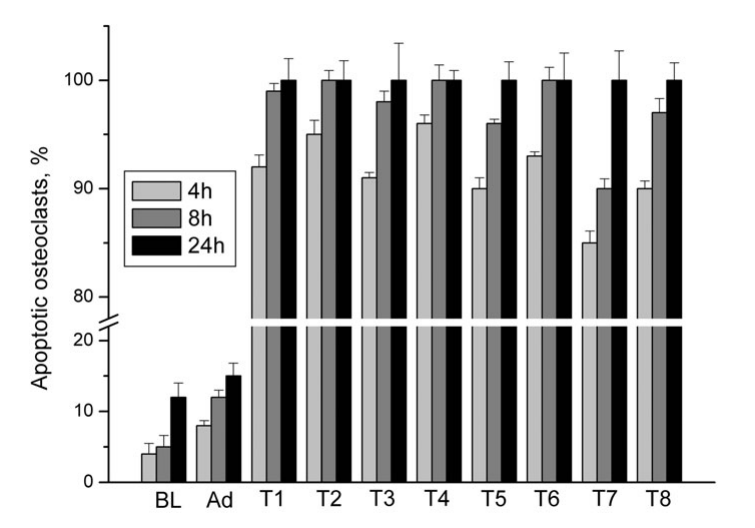
**Apoptosis index in osteoclast cultures. **Cells were treated with transfection reagents for 2 h, followed by culture for 4 h, 8, or 24 h. Nuclei were stained with Hoechst and apoptotic osteoclasts were counted with a fluorescence microscope. BL, baseline with no additions; Ad, adenoviral infection of GFP; T1-T8, transfection reagents as shown in Tables 1 and 2. ANOVA: p < 0,001

Cell death is the major problem encountered when using liposomal transfection systems. Therefore we counted the number of apoptotic cells from Hoechst staining using a conventional fluorescence microscope. Cultured osteoclasts were incubated with the transfection reagents for 2 h, followed by a 4 h, 8 h or 24 h culture period. In the baseline control, where no transfection reagents or adenoviruses were added, only some apoptotic nuclei were found and multinuclear osteoclasts remained polarized and active, as determined by actin ring morphology (Figure 1, [18]) and resorption activity measurements (Figures 2 and 3). When samples treated with the transfection reagents were evaluated, large numbers of apoptotic nuclei were seen and only some nuclei remained unfractionated (Figure 4). Intact osteoclasts could not be found in these samples, and resorption activity was totally lost. The lack of a dose-response suggests that even smaller amounts of liposomes or PEI were not tolerable to the osteoclasts. Some apoptotic nuclei were also seen in the adenovirus-treated samples, but the majority of the nuclei remained intact and many osteoclasts remained actively resorbing bone.

### Viability assay

**Figure 5:**
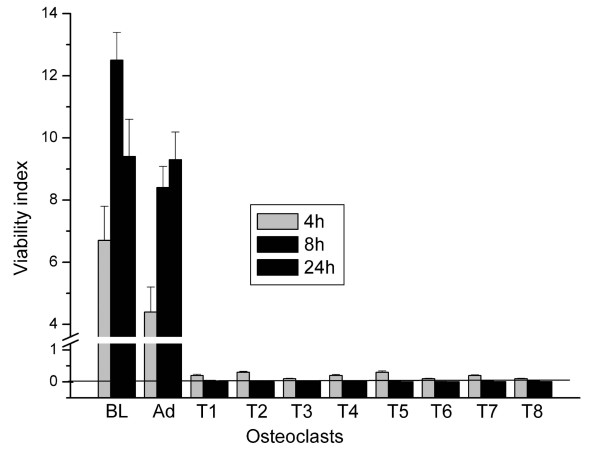
**Viability index in osteoclast cultures. **Cells were treated with transfection reagents for 2 h, followed by culture for 4 h, 8, or 24 h. Osteoclast differentiation cultures were performed on collagen-coated plates to allow the use of the microplate reader. After transfection, cells were stained with Calcein AM and EthD and fluorescence of the dyes was measured using appropriate band pass filters. BL, baseline with no additions; Ad, adenoviral infection of GFP; T1-T8, transfection reagents as shown in Tables 1 and 2. ANOVA: p < 0,001

In order to determine whether any combination of transfection reagent concentration and incubation time would allow cell survival, we cultured osteoclasts on 96 well plates and measured dead and live cell fluorescence with a microplate reader. As can be seen from Figure 5, we could not avoid killing cells with the transfection reagents. When the samples were monitored in more detail after cytochemical staining for the osteoclast marker enzyme TRACP [19], it became evident that a total loss of osteoclasts occurred already after a 1 h treatment with transfection reagents. Adenoviral gene delivery also resulted in osteoclast death and decreased viability, but the majority of the cells remained alive and many cells expressed the transgene.

**Figure 6:**
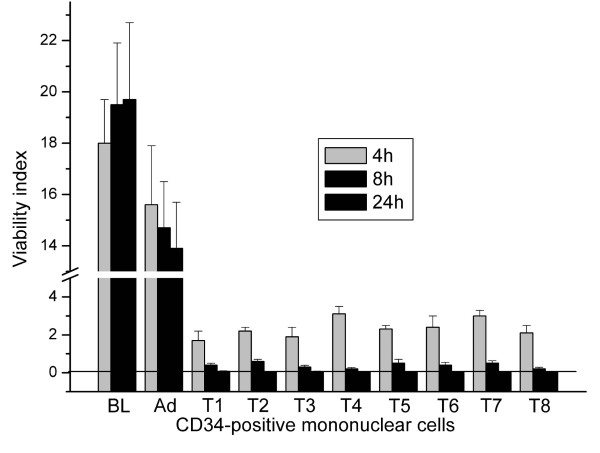
**Viability index in CD34-positive mononuclear cell cultures. **Cells were treated with transfection reagents for 2 h, followed by culture for 4 h, 8, or 24 h. CD34-positive cells were grown on collagen-coated plates and after transfection, cells were stained with Calcein AM and EthD. Fluorescence of the dyes was measured using the microplate reader and appropriate filter sets. BL, baseline with no additions; Ad, adenoviral infection of GFP; T1-T8, transfection reagents as shown in Tables 1 and 2. ANOVA: p < 0,001

We also wanted to check if it would be possible to transfect the non-adherent CD34-positive mononuclear cells and then induce osteoclast differentiation. The Live-Dead assay was thus performed also with the mononuclear precursor cells. As can be seen from Figure 6, the viability indexes remained somewhat higher but far too low as compared to the baseline control or to the adenovirus-treated samples.

### Transfection efficiency

GFP expression was followed in adherent osteoclasts and in non-adherent mononuclear precursors transfected for 4 hours and cultured in fresh medium for 1 h, 24 h, 48 h or 5 days. No GFP expression was noticed in osteoclasts after transfection with any of the transfection systems tested (Table 1). In comparison, adenoviral delivery of the transgene resulted in a 15% transfection efficiency of multinuclear osteoclasts. When CD34-positive non-adherent precursor cells were transfected, some cells were positive 24 h and 48 h after transfection, but no positive cells were seen on day 5 with any of the transfection reagents tested (Table 2). In the adenovirus-infected cultures, multiple GFP expressing cells was seen 24 h and 48 h after infection and some cells also 5 days after infection. These data suggest that transfection of the osteoclasts or the mononuclear precursor cells was not feasible with the conventional transfection methods.

**Table 1:** Transfection efficiency (% of live cells) in mature osteoclast cultures

	**1 h**	**24 h**	**48 h**	**5 d**
Baseline	0	0	0,12 ± 0,042	0
Adenovirus	1,2 ± 0,095	9,7 ± 1,4	15,2 ± 3,4	3,8 ± 0,96
T1: Metafectene	0	0	0	0
T2: Lipofectamine plus	0	0	0	0
T3: Tfx-50	0	0	0	0
T4: FuGene6	0	0	0	0
T5: DOTAP	0	0	0	0
T6: DOSPER	0	0	0	0
T7: JetPei	0	0	0	0
T8: DuoFect	0	0	0	0

GFP-expressing and negative osteoclasts were counted using fluorescence microscopy and phase optics, and transfection efficiencies were counted. ANOVA: p = 1,4 × 10^-8^, n = 5.

**Table 2:** Transfection efficiency (% of live cells) in CD34-positive mononuclear cell cultures

	**1 h**	**24 h**	**48 h**	**5 d**
Baseline	0	0,13 ± 0,031	0,47 ± 0,08	0
Adenovirus	2,7 ± 0,12	13,1 ± 1,4	22,6 ± 3,4	4,2 ± 0,96
T1: Metafectene	0	0,12 ± 0,042	0,23 ± 0,053	0
T2: Lipofectamine plus	0	0,24 ± 0,060	0,30 ± 0,11	0
T3: Tfx-50	0	0,10 ± 0,037	0,41 ± 0,091	0
T4: FuGene6	0	0,23 ± 0,071	0,22 ± 0,13	0
T5: DOTAP	0	0	0,56 ± 0,064	0
T6: DOSPER	0	0,16 ± 0,046	0,28 ± 0,13	0
T7: JetPei	0	0,20 ± 0,050	0,50 ± 0,15	0
T8: DuoFect	0	0	0,12 ± 0,056	0

GFP-expressing and negative osteoclasts were counted using fluorescence microscopy and phase optics, and transfection efficiencies were counted. ANOVA: p = 2,3 × 10^-11^, n = 5.

## Discussion

Osteoclasts are cells that need to be cultured as primary cells or as a differentiation culture from bone marrow-derived mononuclear precursor cells. The natural substrate of osteoclasts is bone, and seeding the cells on a non-natural substrate, like plastic or glass, has a major effect on the regulation of gene expression and cell morphology [18,20]. Therefore we aimed at transfecting multinuclear osteoclasts adhered to bovine cortical bone, a widely used system in osteoclast research. Adenoviral transfection of osteoclasts was used in this study as the reference gene transfer system, while it has been shown to work also with osteoclasts [15]. CAR-receptor bound adenoviruses are internalized via endocytosis after attachment to α_v _integrins, which are widely distributed on the osteoclast surface. Although viral gene delivery is at it's best very efficient and rapid, a strong promoter may drive excessive transgene production and interfere with normal cell physiology. The use of human pathogens, like adeno- and lentiviruses, also requires special attention and authorization, while conventional transfection methods can be used in any laboratory.

Commercial modifications of liposomal gene delivery systems and PEI-dependent endosomal disruption systems were systematically evaluated to determine whether any of the concentration-incubation time combinations would result in osteoclast transfection. To our disappointment, however, none of the 8 transfection systems could provide satisfactory osteoclast transfection efficiency. GFP-tagged actin was used as the transgene for easy monitoring of gene transfer, but no transfected osteoclasts were noticed. Adenoviral gene delivery was the only method capable of providing sufficient transfection efficiency. Among the non-adherent mononuclear precursor cells, an equally poor transfection rate was obtained. The most striking effect was the vast induction of apoptosis with both cationic liposomes and with PEI-dependent endosomal proton sponges. When uptake of the transfection reagent-packed DNA into the cells was monitored in more detail, it could be noted that most of the molecules never penetrated the plasma membrane. It was recently shown that cell-surface glycosaminoglycans are capable of inhibiting transfection [8]. The osteoclast plasma membrane is coated with large amounts of hyaluronic acid and other glycoproteins (for review see [21]), and this may explain why the transfection reagents are unable to deliver their cargo to the plasma membrane. Another explanation for the lack of transfection may be the low cell density. While commercial transfection reagents are suggested to be used in sub-confluent to confluent cell cultures, our osteoclast cultures were appr. 50% confluent (Figure 1d). Our cells were non-dividing, and this may also contribute to the transfection difficulties.

Mature osteoclasts cannot be grown as suspension cultures and confluency is difficult to control. However, osteoclasts take up plasma membrane-impermeable DNA- and RNA molecules from culture medium [22-24]. For antisense and siRNA-research, it would be optimal to increase the uptake and intracellular availability of gene knockdown-molecules in osteoclast cultures. While viral gene transfer is difficult to control, the primary choice for gene knockdown experiments would be a non-viral system that allows transgene packaging, protection and sufficient bioavailability.

## Conclusion

Although many cell lines and some primary cells are easy to transfect using calcium phosphate, DEAE-dextran, electroporation, scrape loading or liposomal transfection systems, these systems cannot be used on multinuclear osteoclasts. These large, adherent, non-dividing cells are fragile and undergo apoptosis rapidly when challenged chemically or mechanically. Optimal cells for commercial transfection systems should be in sub-confluent, rapidly dividing growth phase, which cannot be provided in osteoclast cultures. Microinjection may be used for osteoclast transfection, if only a few transfected osteoclasts are enough and the expertise is available. For proper transfection of higher numbers of osteoclasts, however, the only rational tools are the viral delivery systems.

## Materials and methods

### Cell culture

Human bone marrow-derived CD34-positive mononuclear cells were cultured on bovine cortical bone slices in the presence of M-CSF (33 ng/ml, R&D Systems, UK) and RANKL (66 ng/ml, Peprotech, UK) as suggested by the supplier (Cambrex, USA). TGF-β1 (1 ng/ml, R&D Systems, UK) was added on day 3, and adherent, terminally differentiated osteoclasts were transfected on day 7. When non-adherent osteoclast precursors were used, the transfections were performed on day 1. Cells were cultured in high-glucose DMEM supplemented with 10% heat-inactivated fetal calf serum, 20 mM HEPES, 100 U/ml penicillin and 100 mg/ml streptomycin (all from Gibco Invitrogen, UK). Cells were grown in 96 well plates with 200 μl of medium for fluorescence measurements with a plate reader. Bovine cortical bone slices were 150-180 μm thick transversal sections that were sonicated and sterilized by dipping in 70% ethanol before use. A control group of cells attached to glass coverslips coated with type I collagen (BD Biosciences, Belgium) was also included. Non-attached cells were transfected in wells containing type I collagen-coated glass coverslips or bone slices.

### Transfection systems

The plasmid containing EGFP-actin (Clontech, USA) was transfected to the cells to allow fluorescent visualization of transfected actin filaments. For liposome-mediated transfection, Metafectene (Biontex, USA), Lipofectamine Plus (Gibco Invitrogen, UK), Tfx-50 (Promega Corp, USA) and FuGene6, DOTAP and DOSPER (all from Roche, Germany) were used according to the supplier's instructions. Reagent/DNA ratios were as follows: 1 μg plasmid DNA was complexed with 1.5, 3.0 or 6.0 μl of FuGene6 or Lipofectamine Plus transfection reagent; or with 2.0, 3.0 or 4.0 μl of Tfx-50 or Metafectene transfection reagent; or with 5, 7.5 or 10 μg of DOTAP; or with 3, 7.5 or 12 μg of DOSPER. Also the endosomal disruption-based transfection systems JetPei (PolyTransfection, USA) and DuoFect (Quantum Appligene, USA) were used according to the manufacturer's instructions. For DuoFect transfection, 50 μM deferrioxamine was added to the culture medium 24 h before transfection. With these systems, 1 μg plasmid DNA was complexed with 0.5, 0.75 or 1.0 μl of DuoFect transfection reagent or with 1.5, 3 or 4.5 μl of JetPei transfection reagent.

To test the optimal transfection reagent-to-DNA ratio, cells were incubated with transfection reagents for 2 h the presence of serum, dipped in warm PBS and transferred onto fresh culture plates containing medium and osteoclast growth factors for an additional culture period of 48 h. Cell morphology and transgene expression were monitored microscopically and the following reagent-to-DNA ratios were chosen to be used in the future experiments: 1 μg plasmid DNA was complexed with 3.0 μl of FuGene6, Lipofectamine Plus, Tfx-50 or Metafectene transfection reagent; or with 7.5 μg of DOTAP or DOSPER; or with 1.0 μl of DuoFect; or with 4.5 μl of JetPei transfection reagent. In the following experiments, cells were incubated with transfection reagents for 2 h in the presence of serum, dipped in warm PBS and transferred onto fresh culture plates containing medium and osteoclast growth factors for an additional culture period of 4 h, 8 h or 24 h. Transgene expression and cell viability were evaluated with help of a fluorescence microscope (Leica) and a microplate reader (Victor2, Wallac).

A commercial adenovirus resulting in the expression of GFP under the CMV promoter was used as the transfection control (QBiogene, USA). Cells were infected with 5000 virus particles of Ad5.CMV-GFP in 100 μl medium for 1 h, after which 100 μl of fresh medium and osteoclast growth factors were added. GFP expression and cell viability was evaluated as already described.

### Transfection efficiency and viability

Transgene expression in the cells was monitored under fluorescence microscope 1 h, 24 h, 48 h and 5 days after transfection, and all GFP-positive mononuclear cells and osteoclasts (cells with at least 3 nuclei) were counted. For counting apoptotic cells, 3% paraformaldehyde-2% sucrose was used for fixing the cells prior to staining nuclei with Hoechst as suggested by the supplier (Molecular Probes, USA). Apoptotic nuclei were counted under fluorescence microscope. To monitor cell viability in detail, we stained dead and live cells with the Live/Dead-system (Molecular Probes, USA). Cells grown on 96 well plates were stained after transfection by adding 7 μM Calcein AM (stained live cells) and 5 μM ethidium homodimer-1 (EthD, detected dead cells) to the cell cultures that were washed with warm PBS. Cells were incubated with the dyes for 45 min in 100 μl PBS, followed by fluorescence intensity measurements using exitation/emission filter sets of 495/520 nm (Calcein AM) and 530/642 nm (EthD). Viability indexes were counted by dividing the live cell fluorescence by the dead cell fluorescence.

### Morphological analysis

The effects of transfection reagents on the morphology of cultured cells were monitored during culture with phase optics, and more detailed morphological analysis was performed on fixed samples. Cells were fixed in 3%PFA-2% sucrose for 15 min. To monitor confluency and osteoclast formation capacity in the cultures, cells were fixed and stained for TRACP with the Leukocyte Acid Phosphatase kit (Sigma, USA). Bone resorbing osteoclasts were determined by actin ring staining with AlexaFluor^488 ^Phalloidin (Molecular Probes, USA). Resorption activity was monitored in the samples by biotinylating the existing resorption pits immediately before transfection with sulfo-NHS-biotin (Pierce, USA) as described before [25]. After transfection and further culture, samples were fixed and biotin was detected with FITC-streptavidin (DAKO, Denmark) and all resorption pits were stained with TRITC-WGA lectin (Sigma Aldrich, USA).

### Statistical analysis

Data are expressed as mean ± SD of four replicas and all experiments were independently performed twice (n = 8). Differences from the control were examined for statistical significance by analysis of variance and student's T-test. A p-value less than 0,05 was considered significant.

## Authors' contributions

TLL is responsible for the content of this article.
